# 4-(Di­phenyl­meth­oxy)-3-eth­oxy­benzaldehyde

**DOI:** 10.1107/S2414314621003564

**Published:** 2021-04-09

**Authors:** Erika Samoľová, Aliakbar Dehno Khalaji, Václav Eigner

**Affiliations:** a Institute of Physics AS CR, v.v.i., Na Slovance 2, 182 21 Prague 8, Czech Republic; b Department of Chemistry, Faculty of Science, Golestan University, Gorgan, Iran; University of Aberdeen, Scotland

**Keywords:** Schiff bases, crystal structure, weak inter­molecular inter­actions

## Abstract

In the title compound, C_22_H_20_O_3_, the dihedral angle between the aromatic rings linked by the methine group is 81.265 (4)° and the eth­oxy side chain adopts an extended conformation [C—O—C—C = 177.24 (10)°]. In the crystal, weak C—H⋯π and C—H⋯O inter­actions link the mol­ecules into sheets.

## Abstract 2

The packing of the title compound features weak C—H⋯O and C—H⋯π inter­actions.

## Graphical abstract

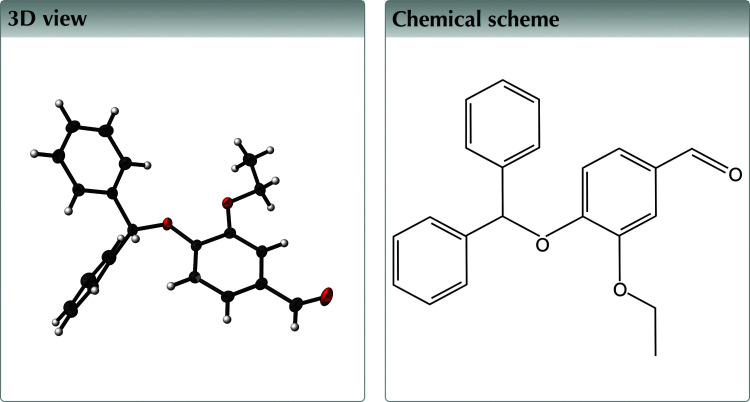

## Structure description

**Figure 1:**
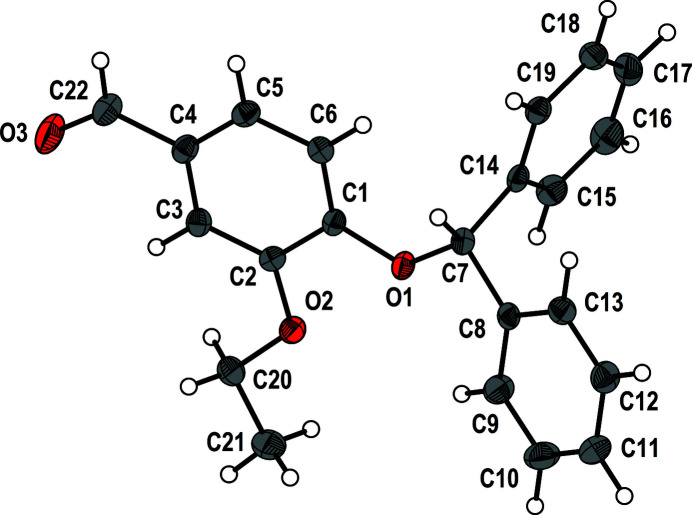
The mol­ecular structure of the title compound with displacement ellipsoids drawn at the 50% probability level.

The preparation of Schiff bases (Omidi & Kakanejadifard, 2020 ▸) is nowadays an inter­esting topic, because of their various application and properties (*e.g.*, Kizilkaya *et al.*, 2020 ▸). As part of our studies in this area, the title aldehyde, C_22_H_20_O_3_, was prepared as a precursor to new Schiff bases and we now describe its crystal structure (Fig. 1 ▸).

**Figure 2:**
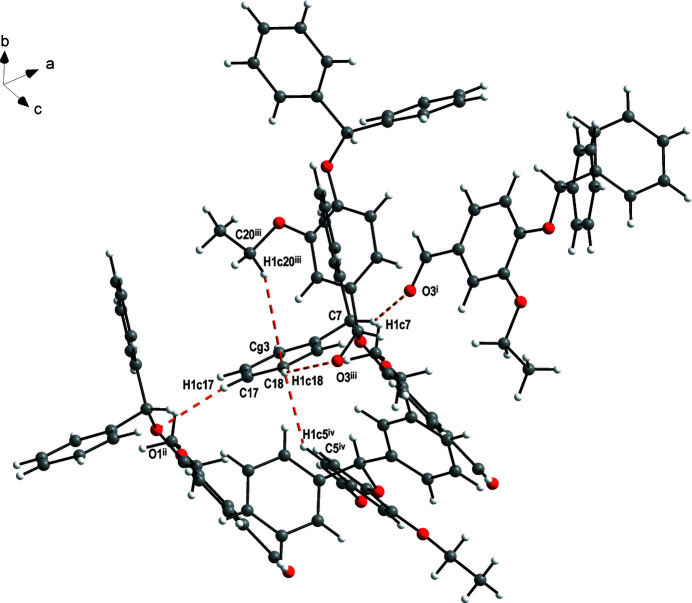
Partial packing diagram showing the hydrogen bonds in the title compound. Hydrogen atoms not involved in hydrogen bonding were omitted for clarity. Symmetry codes: (i) −*x* + 1, *y* + 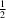 , −*z* + 1; (ii) *x* − 1, *y*, *z*; (iii) −*x* + 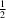 , *y* + 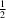 , *z*.

As expected, the C2—O2, C20—O2, C1—O1 and C7—O1 bond lengths reveal single bond character while C22=O3 is a double bond. The dihedral angle between the C1–C6 and C8–C13 aromatic rings connected by the C7 methine group is 81.265 (4)°. In the crystal, weak C—H⋯O (Table 1 ▸) and C—H⋯π inter­actions [C20^iii^—H1*c*20^iii^⋯*Cg*3 = 3.05 Å and C5^iv^—H1*c*5^iv^⋯*Cg*3 = 2.89 Å; symmetry codes: (iii) 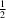 − *x*, 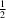 + *y*, *z*; (iv) *x* − 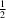 , *y*, 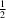 − *z*; *Cg*3 is the centroid of the C14–C19 ring] are observed. These link the mol­ecules into sheets lying perpendicular to the *c*-axis direction (Fig. 2 ▸).

## Synthesis and crystallization

3-Eth­oxy-4-hy­droxy benzaldehyde (0.20 mmol) and potassium carbonate (0.40 mmol) were mixed in di­methyl­formamide (25 ml) and stirred for 0.5 h. A solution of di­phenyl­bromo­methane (0.2 mmol) in ethanol (20 ml) was added dropwise and the mixture was stirred at 80°C for 24 h. After that, the solution was concentrated under reduced pressure. The cream precipitate of the title compound formed by adding cold water (250 ml) was filtered off and washed several times with cold ethanol. Colourless slabs were recrystallized from the mixed solvents of chloro­form and ethanol (1:1).

## Refinement

Crystal data, data collection and structure refinement details are summarized in Table 2 ▸.

## Supplementary Material

Crystal structure: contains datablock(s) global, I. DOI: 10.1107/S2414314621003564/hb4378sup1.cif

Structure factors: contains datablock(s) I. DOI: 10.1107/S2414314621003564/hb4378Isup2.hkl

CCDC reference: 2075001

Additional supporting information:  crystallographic information; 3D view; checkCIF report

## Figures and Tables

**Table 1:** Hydrogen-bond geometry (Å, °)

*D*—H⋯*A*	*D*—H	H⋯*A*	*D*⋯*A*	*D*—H⋯*A*
C7—H1*c*7⋯O3^i^	0.96	2.52	3.3794 (15)	148
C17—H1*c*17⋯O1^ii^	0.96	2.57	3.4440 (15)	152
C18—H1*c*18⋯O3^iii^	0.96	2.53	3.2906 (16)	136

Symmetry codes: (i) 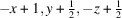 ; (ii) 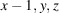 ; (iii) 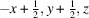 .

**Table 2:** Experimental details

Crystal data	
Chemical formula	C_22_H_20_O_3_
*M*_r_	332.4
Crystal system, space group	Orthorhombic, *P* *b* *c* *a*
Temperature (K)	120
*a*, *b*, *c* (Å)	8.1123 (4), 15.8713 (9), 27.6155 (14)
*V* (Å^3^)	3555.6 (3)
*Z*	8
Radiation type	Mo *K*α
μ (mm^−1^)	0.08
Crystal size (mm)	0.83 × 0.32 × 0.26

Data collection	
Diffractometer	Rigaku Oxford Diffraction Xcalibur, AtlasS2, Gemini ultra
Absorption correction	Analytical (*CrysAlis PRO*; Rigaku OD, 2015 ▸)
*T*_min_, *T*_max_	0.958, 0.982
No. of measured, independent and observed [*I* > 3σ(*I*)] reflections	16215, 4454, 3284
*R*_int_	0.028
(sin θ/λ)_max_ (Å^−1^)	0.695

Refinement	
*R*[*F* ^2^ > 2σ(*F* ^2^)], *wR*(*F* ^2^), *S*	0.040, 0.107, 1.44
No. of reflections	4454
No. of parameters	227
H-atom treatment	H-atom parameters constrained
Δρ_max_, Δρ_min_ (e Å^−3^)	0.22, −0.20

Computer programs: *CrysAlis PRO* (Rigaku OD, 2015 ▸), *SUPERFLIP* (Palatinus & Chapuis, (2007 ▸), *JANA2006* (Petříček *et al.*, 2014 ▸) and *DIAMOND* (Brandenburg, 1999 ▸).

## full crystallographic data

### Crystal data

**Table:**

C_22_H_20_O_3_	*F*(000) = 1408
*M**_r_* = 332.4	*D*_x_ = 1.242 Mg m^−^^3^
Orthorhombic, *P**b**c**a*	Mo *K*α radiation, λ = 0.71073 Å
Hall symbol: -P -2xab;-2ybc;-2zac	Cell parameters from 4539 reflections
*a* = 8.1123 (4) Å	θ = 3.9–29.2°
*b* = 15.8713 (9) Å	µ = 0.08 mm^−^^1^
*c* = 27.6155 (14) Å	*T* = 120 K
*V* = 3555.6 (3) Å^3^	Plate, colourless
*Z* = 8	0.83 × 0.32 × 0.26 mm

### Data collection

**Table:**

Rigaku Oxford Diffraction Xcalibur, AtlasS2, Gemini ultra diffractometer	4454 independent reflections
Radiation source: X-ray tube	3284 reflections with *I* > 3σ(*I*)
Graphite monochromator	*R*_int_ = 0.028
Detector resolution: 5.1783 pixels mm^-1^	θ_max_ = 29.6°, θ_min_ = 3.6°
ω scans	*h* = −11→8
Absorption correction: analytical (CrysalisPro; Rigaku OD, 2015)	*k* = −20→20
*T*_min_ = 0.958, *T*_max_ = 0.982	*l* = −37→37
16215 measured reflections	

### Refinement

**Table:**

Refinement on *F*^2^	H-atom parameters constrained
*R*[*F*^2^ > 2σ(*F*^2^)] = 0.040	Weighting scheme based on measured s.u.'s *w* = 1/(σ^2^(*I*) + 0.0016*I*^2^)
*wR*(*F*^2^) = 0.107	(Δ/σ)_max_ = 0.0004
*S* = 1.44	Δρ_max_ = 0.22 e Å^−^^3^
4454 reflections	Δρ_min_ = −0.20 e Å^−^^3^
227 parameters	Extinction correction: B-C type 1 Gaussian isotropic [Becker, P. J. & Coppens, P. (1974). *Acta Cryst.* A30, 129–147]
0 restraints	Extinction coefficient: 13000 (2000)
80 constraints	

### Special details

**Table:**

Refinement. All hydrogen atoms were discernible in difference Fourier maps and could be refined to reasonable geometry, but according to common practice they were refined as riding atoms with C—H = 0.96 Å and *U*_iso_(H) = 1.2*U*_eq_(C).

### Fractional atomic coordinates and isotropic or equivalent isotropic displacement parameters (Å^2^)

**Table:**

	*x*	*y*	*z*	*U*_iso_*/*U*_eq_	
O1	0.34686 (10)	0.62710 (5)	0.13059 (3)	0.0193 (2)	
O2	0.52510 (10)	0.49847 (5)	0.11100 (3)	0.0203 (2)	
O3	0.64458 (13)	0.36127 (6)	0.27937 (3)	0.0385 (3)	
C1	0.38844 (13)	0.57930 (7)	0.16975 (4)	0.0170 (3)	
C2	0.48965 (13)	0.50922 (7)	0.15898 (4)	0.0170 (3)	
C3	0.54565 (14)	0.45894 (7)	0.19596 (4)	0.0195 (3)	
C4	0.50097 (14)	0.47681 (7)	0.24410 (4)	0.0205 (3)	
C5	0.40159 (14)	0.54531 (8)	0.25426 (4)	0.0206 (3)	
C6	0.34409 (14)	0.59647 (8)	0.21712 (4)	0.0198 (3)	
C7	0.27297 (14)	0.70813 (7)	0.13914 (4)	0.0171 (3)	
C8	0.32021 (13)	0.76454 (7)	0.09687 (4)	0.0178 (3)	
C9	0.39897 (15)	0.73419 (8)	0.05577 (4)	0.0261 (4)	
C10	0.43962 (18)	0.78854 (9)	0.01835 (5)	0.0326 (4)	
C11	0.40310 (16)	0.87355 (9)	0.02180 (5)	0.0297 (4)	
C12	0.32477 (15)	0.90430 (9)	0.06254 (5)	0.0261 (4)	
C13	0.28374 (14)	0.85001 (8)	0.10003 (4)	0.0218 (4)	
C14	0.08714 (14)	0.70028 (7)	0.14420 (4)	0.0177 (3)	
C15	−0.00177 (15)	0.65479 (8)	0.11019 (5)	0.0256 (4)	
C16	−0.17290 (16)	0.65229 (9)	0.11241 (5)	0.0322 (4)	
C17	−0.25477 (16)	0.69522 (8)	0.14866 (5)	0.0298 (4)	
C18	−0.16734 (15)	0.73910 (8)	0.18332 (5)	0.0250 (4)	
C19	0.00414 (14)	0.74156 (7)	0.18104 (4)	0.0199 (3)	
C20	0.63310 (15)	0.43012 (8)	0.09834 (4)	0.0245 (4)	
C21	0.64587 (17)	0.42893 (9)	0.04403 (5)	0.0316 (4)	
C22	0.55991 (16)	0.42412 (8)	0.28389 (5)	0.0275 (4)	
H1c3	0.615307	0.411587	0.188954	0.0233*	
H1c5	0.37214	0.557585	0.287186	0.0248*	
H1c6	0.273891	0.643544	0.22428	0.0237*	
H1c7	0.312056	0.732024	0.168939	0.0205*	
H1c9	0.425323	0.675383	0.053266	0.0313*	
H1c10	0.493341	0.767159	−0.010079	0.0391*	
H1c11	0.432179	0.911071	−0.004095	0.0357*	
H1c12	0.298754	0.963157	0.06492	0.0313*	
H1c13	0.22977	0.871597	0.128373	0.0261*	
H1c15	0.055107	0.624903	0.085003	0.0307*	
H1c16	−0.23405	0.620772	0.088795	0.0386*	
H1c17	−0.373047	0.694527	0.149741	0.0357*	
H1c18	−0.224467	0.767824	0.208906	0.0301*	
H1c19	0.065108	0.772039	0.205141	0.0239*	
H1c20	0.586452	0.377955	0.10939	0.0294*	
H2c20	0.740136	0.439896	0.112063	0.0294*	
H1c21	0.719041	0.384571	0.034222	0.0379*	
H2c21	0.538733	0.419448	0.030313	0.0379*	
H3c21	0.687979	0.482033	0.032892	0.0379*	
H1c22	0.529438	0.440345	0.316162	0.033*	

### Atomic displacement parameters (Å^2^)

**Table:**

	*U*^11^	*U*^22^	*U*^33^	*U*^12^	*U*^13^	*U*^23^
O1	0.0248 (4)	0.0172 (4)	0.0158 (4)	0.0056 (3)	−0.0024 (3)	0.0000 (3)
O2	0.0249 (4)	0.0180 (4)	0.0180 (4)	0.0037 (3)	0.0006 (3)	−0.0005 (3)
O3	0.0567 (6)	0.0310 (6)	0.0277 (5)	0.0151 (5)	−0.0056 (4)	0.0064 (4)
C1	0.0164 (5)	0.0162 (6)	0.0185 (6)	−0.0025 (5)	−0.0032 (4)	0.0020 (4)
C2	0.0181 (5)	0.0164 (6)	0.0165 (5)	−0.0037 (5)	−0.0010 (4)	−0.0009 (4)
C3	0.0207 (6)	0.0160 (6)	0.0217 (6)	−0.0001 (5)	−0.0018 (5)	0.0006 (4)
C4	0.0219 (6)	0.0190 (6)	0.0205 (6)	−0.0036 (5)	−0.0029 (5)	0.0032 (5)
C5	0.0220 (6)	0.0219 (6)	0.0180 (6)	−0.0037 (5)	−0.0007 (5)	0.0006 (5)
C6	0.0188 (5)	0.0197 (6)	0.0208 (6)	0.0006 (5)	0.0000 (4)	−0.0005 (5)
C7	0.0197 (6)	0.0151 (6)	0.0164 (5)	0.0019 (5)	−0.0028 (4)	−0.0022 (4)
C8	0.0143 (5)	0.0208 (6)	0.0182 (5)	−0.0004 (5)	−0.0042 (4)	0.0004 (4)
C9	0.0328 (7)	0.0224 (7)	0.0230 (6)	0.0036 (6)	0.0031 (5)	0.0003 (5)
C10	0.0405 (8)	0.0327 (8)	0.0245 (7)	0.0045 (6)	0.0101 (6)	0.0017 (6)
C11	0.0326 (7)	0.0299 (7)	0.0267 (7)	−0.0012 (6)	0.0039 (5)	0.0093 (6)
C12	0.0280 (6)	0.0200 (7)	0.0304 (7)	0.0006 (5)	−0.0002 (5)	0.0033 (5)
C13	0.0218 (6)	0.0211 (6)	0.0225 (6)	0.0012 (5)	0.0008 (5)	−0.0010 (5)
C14	0.0190 (5)	0.0145 (6)	0.0195 (6)	−0.0008 (5)	−0.0033 (4)	0.0050 (4)
C15	0.0268 (7)	0.0270 (7)	0.0230 (7)	−0.0037 (5)	−0.0032 (5)	−0.0009 (5)
C16	0.0279 (7)	0.0352 (8)	0.0335 (8)	−0.0112 (6)	−0.0109 (6)	0.0019 (6)
C17	0.0178 (6)	0.0313 (7)	0.0402 (7)	−0.0048 (6)	−0.0016 (5)	0.0120 (6)
C18	0.0234 (6)	0.0210 (6)	0.0307 (7)	0.0010 (5)	0.0053 (5)	0.0076 (5)
C19	0.0222 (6)	0.0167 (6)	0.0208 (6)	−0.0010 (5)	−0.0011 (5)	0.0029 (5)
C20	0.0256 (6)	0.0223 (6)	0.0257 (6)	0.0064 (5)	0.0022 (5)	−0.0009 (5)
C21	0.0347 (7)	0.0333 (8)	0.0267 (7)	0.0066 (6)	0.0065 (6)	−0.0034 (6)
C22	0.0347 (7)	0.0257 (7)	0.0221 (7)	0.0013 (6)	−0.0025 (5)	0.0035 (5)

### Geometric parameters (Å, º)

**Table:**

O1—C1	1.3632 (13)	C11—C12	1.3813 (18)
O1—C7	1.4380 (13)	C11—H1c11	0.96
O2—C2	1.3662 (13)	C12—C13	1.3873 (18)
O2—C20	1.4377 (14)	C12—H1c12	0.96
O3—C22	1.2174 (16)	C13—H1c13	0.96
C1—C2	1.4142 (15)	C14—C15	1.3870 (17)
C1—C6	1.3838 (16)	C14—C19	1.3845 (16)
C2—C3	1.3736 (16)	C15—C16	1.3902 (18)
C3—C4	1.4067 (16)	C15—H1c15	0.96
C3—H1c3	0.96	C16—C17	1.3809 (19)
C4—C5	1.3821 (16)	C16—H1c16	0.96
C4—C22	1.4614 (17)	C17—C18	1.3807 (18)
C5—C6	1.3892 (16)	C17—H1c17	0.96
C5—H1c5	0.96	C18—C19	1.3931 (17)
C6—H1c6	0.96	C18—H1c18	0.96
C7—C8	1.5202 (16)	C19—H1c19	0.96
C7—C14	1.5192 (16)	C20—C21	1.5034 (17)
C7—H1c7	0.96	C20—H1c20	0.96
C8—C9	1.3885 (17)	C20—H2c20	0.96
C8—C13	1.3911 (17)	C21—H1c21	0.96
C9—C10	1.3865 (18)	C21—H2c21	0.96
C9—H1c9	0.96	C21—H3c21	0.96
C10—C11	1.3843 (19)	C22—H1c22	0.96
C10—H1c10	0.96		
			
C1—O1—C7	118.07 (8)	C11—C12—H1c12	120.04
C2—O2—C20	117.29 (9)	C13—C12—H1c12	120.04
O1—C1—C2	114.48 (9)	C8—C13—C12	120.52 (11)
O1—C1—C6	125.17 (10)	C8—C13—H1c13	119.74
C2—C1—C6	120.32 (10)	C12—C13—H1c13	119.74
O2—C2—C1	115.12 (9)	C7—C14—C15	119.76 (10)
O2—C2—C3	125.37 (10)	C7—C14—C19	120.76 (10)
C1—C2—C3	119.51 (10)	C15—C14—C19	119.40 (10)
C2—C3—C4	120.02 (10)	C14—C15—C16	120.29 (12)
C2—C3—H1c3	119.99	C14—C15—H1c15	119.86
C4—C3—H1c3	119.99	C16—C15—H1c15	119.86
C3—C4—C5	120.07 (11)	C15—C16—C17	119.88 (12)
C3—C4—C22	120.72 (11)	C15—C16—H1c16	120.06
C5—C4—C22	119.22 (11)	C17—C16—H1c16	120.06
C4—C5—C6	120.37 (11)	C16—C17—C18	120.30 (12)
C4—C5—H1c5	119.81	C16—C17—H1c17	119.85
C6—C5—H1c5	119.81	C18—C17—H1c17	119.85
C1—C6—C5	119.71 (11)	C17—C18—C19	119.70 (11)
C1—C6—H1c6	120.14	C17—C18—H1c18	120.15
C5—C6—H1c6	120.14	C19—C18—H1c18	120.15
O1—C7—C8	107.20 (8)	C14—C19—C18	120.39 (11)
O1—C7—C14	110.82 (9)	C14—C19—H1c19	119.8
O1—C7—H1c7	110.86	C18—C19—H1c19	119.8
C8—C7—C14	111.65 (9)	O2—C20—C21	107.10 (10)
C8—C7—H1c7	110.01	O2—C20—H1c20	109.47
C14—C7—H1c7	106.34	O2—C20—H2c20	109.47
C7—C8—C9	122.61 (10)	C21—C20—H1c20	109.47
C7—C8—C13	118.21 (10)	C21—C20—H2c20	109.47
C9—C8—C13	119.18 (11)	H1c20—C20—H2c20	111.74
C8—C9—C10	120.17 (12)	C20—C21—H1c21	109.47
C8—C9—H1c9	119.91	C20—C21—H2c21	109.47
C10—C9—H1c9	119.91	C20—C21—H3c21	109.47
C9—C10—C11	120.28 (12)	H1c21—C21—H2c21	109.47
C9—C10—H1c10	119.86	H1c21—C21—H3c21	109.47
C11—C10—H1c10	119.86	H2c21—C21—H3c21	109.47
C10—C11—C12	119.93 (12)	O3—C22—C4	125.21 (12)
C10—C11—H1c11	120.04	O3—C22—H1c22	117.39
C12—C11—H1c11	120.04	C4—C22—H1c22	117.4
C11—C12—C13	119.91 (12)		

### Hydrogen-bond geometry (Å, º)

**Table:**

*D*—H···*A*	*D*—H	H···*A*	*D*···*A*	*D*—H···*A*
C7—H1*c*7···O3^i^	0.96	2.52	3.3794 (15)	148
C17—H1*c*17···O1^ii^	0.96	2.57	3.4440 (15)	152
C18—H1*c*18···O3^iii^	0.96	2.53	3.2906 (16)	136

Symmetry codes: (i) −*x*+1, *y*+1/2, −*z*+1/2; (ii) *x*−1, *y*, *z*; (iii) −*x*+1/2, *y*+1/2, *z*.
